# Incidence of bacterial meningitis (2001–2005) in Lazio, Italy: the results of a integrated surveillance system

**DOI:** 10.1186/1471-2334-9-13

**Published:** 2009-02-05

**Authors:** Paolo Giorgi Rossi, Jessica Mantovani, Eliana Ferroni, Antonio Forcina, Elena Stanghellini, Filippo Curtale, Piero Borgia

**Affiliations:** 1 Agency for Public Health, Lazio Region, Rome, Italy; 2 Dipartimento di Economia Finanza e Statistica, University of Perugia, Perugia, Italy

## Abstract

**Background:**

Monitoring the incidence of bacterial meningitis is important to plan and evaluate preventive polices. The study's aim was to estimate the incidence of bacterial meningitis by aetiological agent in the period 2001–2005, in Lazio Italy (5.3 mln inhabitants).

**Methods:**

Data collected from four sources – hospital surveillance of bacterial meningitis, laboratory information system, the mandatory infectious diseases notifications, and hospital information system – were combined into a single archive.

**Results:**

944 cases were reported, 89% were classified as community acquired. *S. pneumoniae *was the most frequent aetiological agent in Lazio, followed by *N. meningitis*. Incidence of *H. influenzae *decreased during the period. 17% of the cases had an unknown aetiology and 13% unspecified bacteria. The overall incidence was 3.7/100,000. Children under 1 year were most affected (50.3/100.000), followed by 1–4 year olds (12.5/100,000). The percentage of meningitis due to aetiological agents included in the vaccine targets, not considering age, is 31%. *Streptococcus spp*. was the primary cause of meningitis in the first three months of life. The capture-recapture model estimated underreporting at 17.2% of the overall incidence.

**Conclusion:**

Vaccine policies should be planned and monitored based on these results. The integrated surveillance system allowed us to observe a drop in *H. influenzae b *meningitis incidence consequent to the implementation of a mass vaccination of newborns.

## Background

Bacterial meningitis, of all infectious diseases, is one of the most relevant for public health professionals and decision makers [1]. There are three main reasons for its relevance: 1) it is a serious disease with a high fatality rate (up to 20%) [2,3] and sequalae [4], although rare, (3.5/100,000) [5] are present and visible in the general population; 2) it affects young children in particular; 3) there are three vaccines against the most common aetiological agents, *Haemophilus influenzae b, Streptococcus pneumoniae*, and *Neisseria meningititis*.

To plan a mass vaccination we need to quantify the portion of the disease that is preventable by vaccination. The information essential for this task is: to identify the cases, to ascertain the aetiology, and to determine if the infection was actually preventable in that patient. Routine surveillance systems, based on notifications of the infectious disease, need to be timely to activate prophylactic control measures and often are not accurate in terms of aetiology, furthermore, they are intrinsically affected by under-reporting [6].

In other words, a sensitive, specific and accurate surveillance system is necessary to plan an efficient mass vaccination.

The solution adopted in several countries has been to enhance notification systems by integrating them with the information collected by different sources, some very sensitive, such as the hospital database, and others very specific, such as laboratory isolates.

A surveillance system that integrates three data sources was implemented in our region in 1997; the initial results of that system have already been described [7,8]. A new source of information, the laboratory information system, was added to it in 1999. The advantages of this integrated surveillance are that we can follow the history of case hospitalizations, including emergency department visits, before and after disease onset, to investigate pre-existing medical conditions and health care outcomes.

This paper summarises the results of 5 years (2001–2005) of bacterial meningitis surveillance in Lazio. We present the incidence of meningitis by aetiological agent (by single bacteria and by vaccine-targeted vaccine targeted bacteria), patient characteristics (age, gender, and place of birth), and by origin of infection (community acquired, hospital and in HIV/AIDS).

## Methods

### Population

Lazio has 5.3 million inhabitants and includes the metropolitan area of Rome (2.9 million); the number of live births per year is about 49.000. We included cases that occurred between 2001 and 2005.

### Sources

Individual data from four different data sources were linked to produce a single database, eliminating redundant records and discrepancies. The following sources of data were analysed:

#### The mandatory Infectious disease notification System (NDS)

The NDS collects reports of acute infectious diseases according to a national law that requires case notification to the Local Health Unit [9]. The notification reports include: personal data of the patients; diagnosis; symptoms onset date; risk factors; and, eventually, *exitus*. Meningococcal diseases (meningitis and sepsis) have to be reported within 12 hours to begin prophylactic measures; meningitis due to other bacteria has to be reported within two days. Notifications are updated after the case is confirmed clinically, and when the outcome of the disease is death.

#### Hospital Surveillance of Bacterial Meningitis

The hospital surveillance system (HSS) of bacterial meningitis [10], in operation since 1994, is a voluntary, partially laboratory-based surveillance system that collects reports of bacterial meningitis from hospitals. Cases are reported to the Agency for Public Health by the medical staff of the hospital health management.

#### Laboratory based surveillance of invasive bacterial diseases

The Laboratory Information System (LIS), which started in 1996 following the enactment of a regional regulation, collects all positive bacterial samples from normally sterile sites [11]. Furthermore, all strains of *Haemophilus influenzae*, *Neisseria meningitidis *and *Streptococcus pneumoniae *must be sent to the central laboratory of the region for serotyping and storage.

#### Hospital Information System

The Hospital Information System (HIS) of the Lazio region has been active since 1995 [7,12]: it routinely collects discharge abstract data from all Lazio public and private hospitals (more than 150 facilities) and includes: admission and discharge dates, discharge status, up to six discharge diagnoses (ICD-9-CM), and up to six hospital procedures (ICD-9-CM).

In addition, we used the Emergency Information System (EIS) to identify the reason for the first admission, in order to determine which were probable hospital infections (see classification of cases). EIS collects records of all emergency room visits in Lazio [13]. It includes all 60 EDs (Emergency Departments) from the region. It reports: the name, date and place of birth of the patient, up to four diagnoses coded according to ICD-9-CM, and the principal reason for the admission. Data from this source were used.

The information about disease outcome was obtained from the HIS (i.e. in-hospital mortality) and NDS (short term follow-up).

### Case selection

We adopted the following inclusion criteria for the four sources:

▪ NDS: all meningitis cases reported with symptom onset in the years 2001–2005;

▪ HSS: all cases reported with symptom onset in the years 2001–2005;

▪ LIS: all positive cerebro-spinal fluid samples with date of diagnosis in the years 2001–2005, for *Neisseria meningitis *we also included blood isolates;

▪ HIS: all hospitalisations reporting a meningitis diagnostic code as principal or secondary diagnoses with admission in the years 2001–2005 (ICD-9-CM codes: 036.0–2, 036.9, 013, 320.0, 320.1, 320.2, 320.3, 320.7, 320.8, 320.9, 003.21). Multiple hospitalizations due to transfers were combined; admissions for transferred patients were followed until discharge or death.

We classified as foreign cases all the cases occurring in foreigners. When residence was not given, we used place of birth.

### Record Linkage

The cases reported from each source were combined to obtain a list of single episodes of meningitis. Linkage keys were name, family name and date of birth for the NDS, LIS and HIS; for the HSS we used age instead of date of birth.

Repeated episodes with the same aetiological agent or compatible aetiological diagnosis (i.e. "non specified"/"specified" and "non specified"/"non specified") were considered as a single episode when they occurred within 30 days of the previous hospital discharge or within 40 days of the onset of symptoms for not-hospitalised cases (30 days + 10 days is the median length of stay for hospitalised cases); otherwise, they were considered as distinct episodes. We checked the clinical records for patients with more than one episode in the study period to ascertain that the second was truly a new episode.

### Origin of the infection

The identified cases of meningitis were classified into three categories according to the origin of infection: probable hospital infection, AIDS/HIV, or community acquired infections. The following criteria were used:

#### Probable hospital infection

the presence of at least one of the following conditions: 1) principal diagnosis other than meningitis or other infectious diseases that could degenerate into meningitis (see appendix); a hospitalisation within 10 days before the onset of symptoms, without a diagnosis of an infectious disease, with a length of stay of more than 24 h; 2) primary or secondary diagnosis of trauma (ICD-9-CM codes 800–959.9) or shunt of the cerebro-spinal fluid (ICD-9-CM codes 996.2, 996.63, 996.75, 997.0, V45.2); 3) diagnosis of trauma or pre-operative admission for surgery reported by the EIS in the emergency room record within 48 h before the onset of symptoms.

#### AIDS/HIV

the presence of an HIV positive or AIDS diagnosis (ICD-9-CM codes 042–043; ICD-9 279.1), in any hospitalisation before or after the onset of meningitis in the HIS 1996–2005.

#### Community acquired

incident episodes of meningitis not classified as probable hospital infection or in AIDS/HIV.

It is important to note that probable hospital infection and AIDS/HIV are not mutually exclusive groups.

### Aetiology

If different data sources reported different specified aetiological agents, the laboratory data was considered correct; when the laboratory record was not available, we checked the clinical records.

We also calculated the proportion of meningitis cases caused by vaccine-targeted bacteria: *Haemophilus influenzae *b, Menigococcus C and the 7 Penumococcus serogroups included in the conjugated vaccine in 0–4 year old children, and the 23 included in the polysaccharide vaccine for 5+ year olds. Furthermore, we also calculated the proportion of vaccine-preventable meningitis in the target ages: for *Haemophilus iinfluenzae *b, Pneumococcus (7 groups) and Meningococcus C 0–2 year old children and 65+ for Pneumoccoccus (23 groups). We decided to include only community-acquired infections in the vaccine-preventable cases. This choice is based on the assumption that most hospital infections, as well as the infections in AIDS/HIV, are due to host factors; and in these cases, the efficacy of vaccination is questionable. This hypothesis also provides a conservative estimate of the vaccine-preventable proportion of meningitis cases.

### Capture-recapture method

Capture-recapture methods are widely used in epidemiology to estimate the size of a population from a multiple-record system [14]. The data set is the contingency table obtained by the cross classification of the observed counts according to whether or not they have been captured by each of the lists. The unknown parameter then is the number of individuals not captured by any of the lists, which can be estimated under reasonable assumptions on how the lists interact. When available, covariates are used to form strata of individuals such that the inclusion probability in each list can be assumed homogeneous. Here we used two different sets of models: the first set of models are based on the assumption that the counts of the contingency table follow a Poisson distribution [15] and belong to the class of log-linear models. To avoid sparse contingency tables, the covariates were considered one at the time. The limitation of this approach lies in the fact that any possible confounding or interactions between different covariates cannot be assessed. Confidence intervals for the undercounts are derived from the property that they follow an asymptotic Gaussian distribution [16]. The second set of models assumes the existence of two latent classes and models the effect of covariates on the odds of belonging to one instead of the other and on the probability of appearing in each latent class conditionally on the covariate [17].

### Ethics

The present study did not require approval from an Ethics Committee. The Agency for Public Health of the Lazio Region is the governmental agency responsible for the collection of infectious disease notifications, hospital discharge records and laboratory surveillance.

The management of these data for public health purposes does not require a patient's informed consent nor does it require any authorization regarding privacy laws.

## Results

### Incidence

944 cases of meningitis were reported by at least one source in the five years of the study period, in 934 individuals (10 people had two independent episodes (table 1)). For 43 other people with two reported episodes, checking the medical records excluded the occurrence of two different episodes of meningitis; consequently the second episode was not considered. The resulting incidence was 3.67/100,000/y in the general population, with a U-shaped curve (table 2): a peak during the first year of life (48.60/100,000/y) and in children aged 1 to 4 years (11.29/100000/y) (table 3), and a second peak in adults (4.26/100,000/y in the >64 year olds). Males had a higher incidence than females (4.27/100,000/y vs 3.12/100,000).

**Table 1:** Number of meningitis cases by aetiology and origin of the infection, 2001–2005, Lazio, Italy.

		Community acquired		Hospital infection		HIV-related	
Aetiological agent	Total	n	%	n	%	n	%
Pneumococcus	221	208	24,6	12	16,2	2	7,7
Meningococcus	161	152	18,0	6	8,1	3	11,5
Tuberculosis	82	66	7,8			16	61,5
Streptococcus spp.*	62	51	6,0	11	14,9		
Staphylococcus spp.	59	48	5,7	10	13,5	1	3,8
Haemophilus	46	43	5,1	3	4,0		
Listeria	28	25	3,0	2	2,7	1	3,8
Salmonella	2	2	0,2				
Other bacteria specified	39	33	3,9	5	6,8	1	3,8
Other bacteria NOS	83	67	7,9	14	18,9	2	7,7
Unknown	161	150	17,8	11	14,9		

**Total**	**944**	**845**	**100,0**	**74**	**100,0**	**26**	**100,0**

* except *Streptococcus pneumoniae*

**Table 2:** Cases of bacterial meningitis, distribution by age and aetiology, 2001–2005, Lazio, Italy.

	0–4	5–9	10–14	15–24	25–64	>64	m.i.	Total
*Population on 1/1/2003*	*230140*	*233211*	*250767*	*540633*	*2942446*	*948608*		*5145805*
								
Pneumococcus	42	3	3	4	99	70		221
Meningococcus	55	12	11	21	56	5	1	161
Tuberculosis	6		1	4	53	18		82
Streptococcus spp.*	29	1	1	2	17	12		62
Stafilococcus spp.	11	4	4	2	26	11	1	59
Haemophilus	25	1		4	6	10		46
Listeria	1			3	14	10		28
Salmonella	1					1		2
Other bacteria specified	11			3	12	13		39
Other bacteria NOS	13	5	5	10	32	18		83
Unknown	25	18	8	16	60	34		161

**Total**	**219**	**44**	**33**	**69**	**375**	**202**	**2**	**944**

* except *Streptococcus pneumoniae*

**Table 3:** Age 0–4 years: quarterly distribution of bacterial meningitis cases by aetiology, 2001–2005, Lazio, Italy.

	1	2	3	4	5	6	7	8	9	10	11	12	13	14	15	16	m.i	Total
Meningococcus	3	9	12	4	5	5	3		1	1	1			3	3	4	1	55
Pneumococcus	1	7	5	3	1	3	3	4	4			1	2	3	1	4		42
Streptococcus spp.*	28		1															29
Haemophilus	2	6	4	1	3	3	2	2				2						25
Stafilococcus spp.	2	1					1			1			2	1	2	1		11
Tuberculosis			1		1		1	1			1					1		6
Listeria	1																	1
Salmonella		1																1
Other bacteria specified	8		1							1							1	11
Other bacteria NOS	3	1	2			1		1								5		13
Unknown	4	2	3		1			1		1	1		2	2	2	5	1	25

**Total**	**52**	**27**	**30**	**8**	**11**	**12**	**10**	**9**	**5**	**4**	**3**	**3**	**6**	**9**	**8**	**20**	**3**	**219**

* except *Streptococcus pneumoniae*

We classified 74 cases as suspected hospital acquired infection (table 1): 45 had been hospitalised in the 10 days before the onset of symptoms, 34 were given a diagnosis of trauma in the emergency room or during the admission. Twenty-six cases were of meningitis in HIV/AIDS, three of these were also hospital-acquired.

Among the 845 community acquired meningitis cases, 695 had an identified aetiology (82.2%); the most common aetiological agent was Pneumococcus (24.6%), the second was Meningococcus (18.0%). The hospital-acquired infections did not show any predominant aetiological bacteria: 40% of the infections were evenly distributed among *Streptococcus spp*., Pneumococcus, *Stafilococcus spp*. Sixty-two percent of HIV/AIDS meningitis was tuberculosis (table 1).

The percentage of unidentified aetiology was 17.1%, the "other not specified bacteria" (ICD-9-CM code 320.7 without the specific code in primary or secondary diagnosis and codes 320.81–320.89) accounted for 12.9%.

### Time trends and geographical patterns

**Figure 1:**
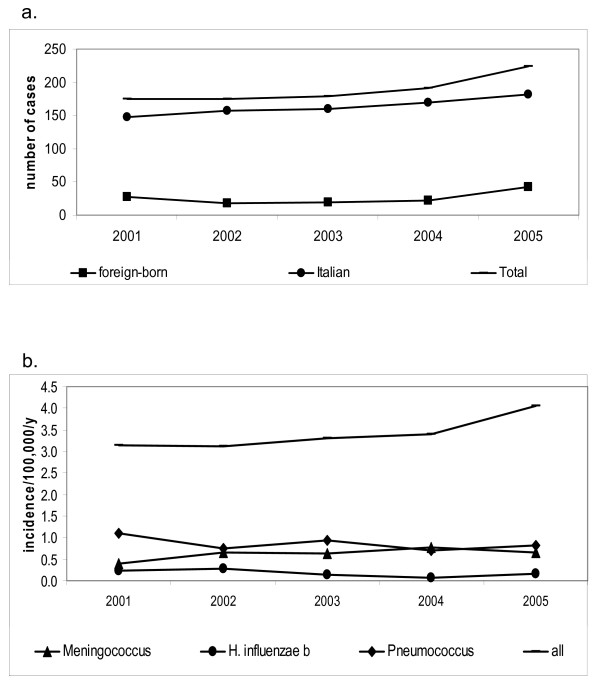
**Bacterial meningitis in Lazio, Italy, 2001–2005**. Number of cases by place of birth and incidence by aetiological agent. 2a, the actual denominator of foreign-born people is not available, so only absolute numbers can be compared; 2b, in the incidence only residents of Lazio are included, independent of citizenship.

The absolute number of meningitis cases showed an increasing trend from 175 to 224 (figure 1a). The increase in incidence is less evident due to a larger denominator in 2005 (from 5.1 to 5.3 million): 3.4/100.000 in 2001 to 4.3/100.000 in 2005 (figure 1b). The increase in the absolute number is stronger for cases in foreigners: from 17 to 38.

The incidence of meningitis due to *H. influenzae *b was the only aetiology that decreased, from 3.49/100,000 to 2.03/100,000 in the 0–4 age bracket.

**Figure 2:**
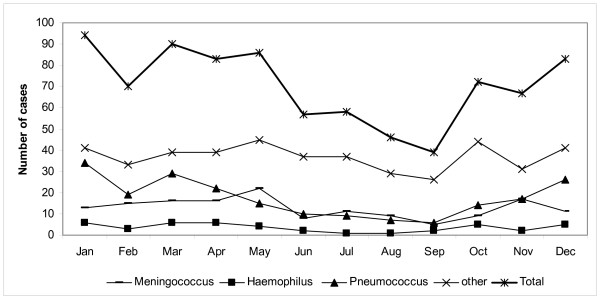
**Bacterial meningitis in Lazio, Italy, 2001–2005**. Seasonality by aetiological agent, for community acquired cases only.

Figure 2 shows the seasonality of community acquired infections. In particular, pneumococcal disease and the others, including unknown aetiology, had a winter peak, while meningococcal disease showed a spring peak.

We observed a statistically significant geographical pattern for community-acquired infections with higher incidence in the city of Rome (standardised incidence rate 114, 95%CI 104–125) and in the suburbs (standardised incidence rate 108, 95%CI 94–124) a lower incidence in rural areas (standardised incidence rate 65, 95%CI 56–76). While there was no discernible geographical trend in hospital infections, 19 out of 26 cases (73%) of AIDS meningitis were residents of Rome.

### Distribution by type of the preventable aetiologies from LIS (table 4)

**Table 4:** Meningitis aetiological agent serotypes identified by the Laboratory Information System, 2001–2005, Lazio, Italy.

Pneumococcus serogroup	n	Meningococcus serogroup	n	Haemophilus serotype	n
		
14	9	B	25	B	5
19	9	C	19	not available	8
23	8	W135	1	Total	13
				
6	8	not available	14		
1	4	Total	59		
				
18	4				
4	3				
7	3				
9	2				
15	2				
22	2				
3	1				
5	1				
8	1				
10	1				
11	1				
12	1				
33	1				
not available	30				
Total	91				

*Haemophilus influenzae *was typed in only 5 cases, all of them were serogroup b. All types of pneumococcal meningitis identified, out of 61 cases, are included in the 23-valent polysaccharide vaccine, while only 70.5% are included in the heptavalent conjugate, in the hypothesis of total cross protection for the serogroups 6, 9, 18, 19 and 23. Out of the 45 *Neisseria meningitis *typed, 19 were serogroup C and one was W-135, both of which are included in the tetra-valent conjugate vaccine. The remaining 25 were serogroup B. The percentage of meningitis types targeted by the vaccine, not considering age, was 31% of the whole burden with a known aetiological agent.

None of the 28 *Streptococcus spp*. that caused meningitis in the first three months of life were typed.

### Fatality rate

**Figure 3:**
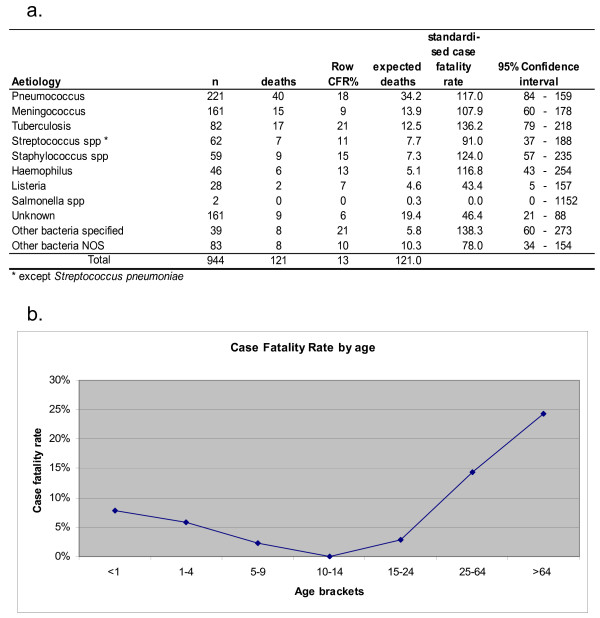
**Bacterial meningitis Case Fatality Rate by aetiological agent and age**. The top of the figure reports the row CFR (%) for each aetiological agent, the expected number of deaths according to age-specific death rates for each age bracket, the standardised fatality ratio (i.e. observed deaths/expected deaths*100) and the relative confidence intervals. The graph reports age-specific fatality rates for all meningitis. The category "other" includes unknown aetiology. 2001–2005, Lazio, Italy.

Only two of the four sources collected information about the death of patient: the HIS, which records in-hospital mortality, and the NDS, which records short-term mortality. We calculated the case fatality rate for the cases reported these two sources. The overall case fatality rate was 13%, the highest was from tuberculosis and other specified bacteria (both with 21%). None of the single aetiologies differed from the mean after adjusting for age; only the unknown aetiology was significantly less fatal than the mean (figure 3a). Hospital infections had a slightly higher fatality rate than community-acquired (15.7% vs. 12.1%), while AIDS/HIV meningitis had a fatality rate of 30.7%.

The fatality rate by age has a J-shaped curve (figure 3b): 7% (9/116) in 0–12 month olds, 6% in 1–4 year olds (6/103), 2% in 5–9 (1/44), 0% in 10–14 (0/33), 3% in 15–24 (2/69), 14% in 25–64 (54/375) and 24% in 65+ (49/202). The case fatality rate was higher for females (14.6% vs 11.1%); the difference is not statistically significant and decreases when adjusted for age.

### Capture re-capture

**Figure 4:**
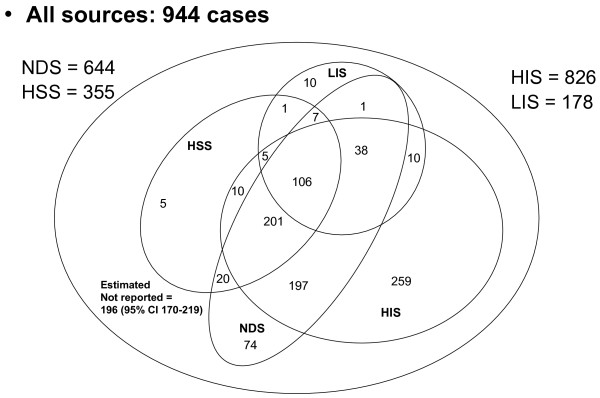
**Cases of bacterial meningitis in Lazio, Italy, 2001–2005**. Results of the record linkage of the four data sources: hospital surveillance of bacterial meningitis (HSS), laboratory information system (LIS), the mandatory infectious diseases notifications (NDS), and hospital information system (HIS). The largest set includes the undetected cases as estimated by the capture-recapture model.

Figure 4 shows the relative contribution of the four sources: the HIS and the NDS identified the most cases (826 and 644 respectively), while the LIS and the HSS identified 178 and 355 cases respectively, of which only 16 were not reported by the other sources.

The latent classes model estimated 17.2% underreporting (some 196 undetected cases 95%CI 176–219). The following covariates reduced the probability of being reported to one of the lists: the presence of AIDS/HIV; probable hospital infections; being a foreigner; and being a foreigner. The underreporting is near zero for meningitis caused by the three bacteria under laboratory surveillance (2.6%) (table 5). These influences were all confirmed in the log linear model.

**Table 5:** Results of the latent classes capture-recapture model to estimate bacterial meningitis under-reporting by patient and disease characteristics.

Variable	Group	total reported	estimated not reported	95% Confidence Interval	% of under-reporting
	All	944	196	176 – 219	17,2
Gender					
	male	526	112	100 – 125	17,5
	female	418	84	76 – 94	16,8
Age					
	0–5	227	37	34 – 41	14,1
	6–64	513	123	110 – 139	19,4
	>64	204	35	32 – 39	14,8
Aetiology					
	Pneumo/Meningo/Haemophilus	428	11	11 – 12	2,6
	other	516	185	164 – 207	26,4
Type of infection					
	AIDS/HIV	26	14	9 – 23	35,2
	Hospital	83	29	22 – 39	25,8
	Community	838	155	140 – 171	15,6
Residence					
	Lazio	857	133	122 – 144	13,4
	out of region	87	63	54 – 75	42,2
Citenzenship					
	Italian	816	114	105 – 123	12,3
	foreign-born	128	82	70 – 95	39,1

2001–2005, Lazio, Italy.

## Discussion

### Limits and methodological remarks

The surveillance system presented here has evolved from the three-source surveillance system presented by Faustini et al in 2001 [18]. We have added the laboratory surveillance, which does not substantially change the sensitivity of the system given the few cases reported by this source alone, but it improved the accuracy of the aetiological information. The previous work on integrated surveillance calculated the specificity of the case definition checking all the case's clinical files; they found that 20% of meningitis reported by the surveillance system was not confirmed by a panel of experts. A similar study was performed in another Italian region with similar results [19].

The surveillance does not include death certificates among the sources, because the mortality information system is not timely and the final version of the archive is available only after a couple of years. Consequently, we cannot ascertain 30-day mortality. A recent study compared the fatality rate of meningitis in Lazio calculated using the information reported by the hospital and the mandatory notification system with the gold standard of linkage with the mortality registry [20], and the results were very similar.

The method we used to distinguish community-acquired meningitis and hospital infections has not been validated. We adapted it from the inclusion criteria for community-acquired pneumonia used by Romano et al in constructing a cohort to evaluate outcomes of patients hospitalised for pneumonia in California [21].

The criteria and the information sources to classify meningitis infection in AIDS/HIV have been used and validated before [12].

### Incidence and trends

The incidence of pneumococcal meningitis in our region is low, but it is comparable to the values found in other European Countries [22] and in another Italian region for children [23]. However, the meningococcal meningitis incidence observed in our region is at the higher end of the range observed in Europe [24-26].

Children experience the highest incidence. There are several aetiologies and the most common, i.e. Pneumococcus, is responsible for only 25%. The percentage of unknown aetiology is comparable to that found with other surveillance systems based on clinical reports and laboratory isolates, but the percentage of "other bacteria" for which we could not identify the aetiology is a concern; these cases are generally detected only by the hospital information system.

There is an increasing trend in the absolute numbers. There were no changes in the surveillance systems in the study period and we tried to exclude any bias due to a reduction of underreporting. The increasing number of meningitis cases is due predominantly to cases that occurred in immigrants. There are three possible causes for this specific increase: a larger denominator, a specific reduction of underreporting in this population, and/or an increased incidence in this group. Despite the fact that there is a substantial lack of knowledge about the foreign population living in our region, over the last three years the number of resident immigrants increased by 14% [27]. Some of the foreign population in recent years have been integrated into Italian society, a process that probably indicates easier access to health services and consequently a higher probability of reporting infectious diseases. Finally, while it cannot be excluded, there is no evidence that meningitis risk has increased in the foreign population in the last five years.

Very few studies have tried to distinguish the burden of community-acquired meningitis and hospital-acquired meningitis, consequently we have little data to use for comparison [28-31].

Capture-recapture models estimate that about 40 cases per year are not reported to any source. Underreporting to the mandatory notification system is estimated to be 50%. The hospital information system is the source that increases the sensitivity of the surveillance. Interestingly, meningitis occurring in foreign citizens is reported less often, reflecting the well-known barriers to health service access experienced by immigrants [32,33].

Other categories of meningitis affected by underreporting are in AIDS patients and the probable hospital infections. The first are probably seen as complications of a disease that has already been reported; while the second are probably affected by an opportunistic underreporting in an effort by the hospital to avoid responsibility. Finally, the absence of underreporting for the three bacteria under specific laboratory surveillance is very encouraging, indicating that the figures on which we are planning our vaccination policies are reliable.

The fatality rate in our setting is 13%, comparable to data reported by other surveillance systems in industrialised countries [26,34]. The rates do not differ by aetiology except for the cases with unknown aetiology that have a significantly lower case fatality rate. A previous validation study found that these cases have a higher proportion of unconfirmed cases [18].

The proportion of cases due to vaccine-targeted bacteria is about 31%, but only one-half of these stuck subjects in the target population of plausible vaccination campaigns, i.e. *Haemophilus influenzae *b, Pneumococcus and Meningococcus in young children or Pneumococcus over 65; consequently, the overall effect of a vaccination campaign would be seen only after several years. Meningitis by *Haemophilus influenzae *b was the only type which decreased in number and incidence, this is probably the effect of increased immunisation coverage, from 30% in 2001 to 89% in 2005. A similar association has been observed in our and other countries [35-38]. More than half of the cases that occurred in the first three months of life are due to *Streptococcus spp*, excluding *Streptococcus pneumoniae*. These kinds of infections are mostly due to mother-child transmission during delivery and are highly preventable with maternal screening and prophylactic antibiotics [39].

## Conclusion

The results of this surveillance system were utilised to define public health control interventions against bacterial meningitis in Lazio and provide baseline data to assess the impact of these measures in the future.

The surveillance system allowed us to monitor the impact of the vaccination campaign against *Haemophylus influenzae *type b. Even though Pneumococcus is the most common pathogen, fewer than 5 cases per year would be prevented by a mass vaccination of infants with the heptavalent conjugate vaccine. A prevention intervention to control streptococcus infections in the first three months of life would have a greater impact on reducing the number of meningitis cases.

## Abbreviations

AIDS/HIV: used in this paper to identify meningitis occurred in person who were AIDS cases or HIV infected; *spp*: used to identify all the species of the genus specified before; NDS: National mandatory Infectious Disease notification System [9]; HSS: Hospital Surveillance System of bacterial meningitis [10]; LIS: Laboratory Information System of the Lazio region [11]; HIS: Hospital Information System of the Lazio region [7,12]; ED: Emergency Department; ICD-9-CM: International Classification of Diseases 9^th ^revision; Clinical Modification; CFR: Case Fatality Rate.

## Competing interests

The Agency for Public Health, Lazio Region has a research grant from Sanofi Pasteur MSD for a different study. The Agency for Public Health of the Lazio Region is the governmental agency responsible for the collection of the infectious disease notifications, hospital discharge records and mortality records, and did not received any financial aid for this study.

## Authors' contributions

PGR designed the study and drafted the manuscript; JM collected all the notifications, performed the record linkage and conducted the statistical analyses except for the capture recapture; EF analysed the case history of hospitalisation and analysed all clinical records; AF and ES performed the capture recapture estimates; FC and PB contributed to the study design and to the manuscript.

## Pre-publication history

The pre-publication history for this paper can be accessed here:

http://www.biomedcentral.com/1471-2334/9/13/prepub
